# Hierarchical structure and chemical composition of complementary segments of the fruiting bodies of *Fomes fomentarius* fungi fine-tune the compressive properties

**DOI:** 10.1371/journal.pone.0304614

**Published:** 2024-06-13

**Authors:** Sophie Klemm, Carsten Freidank-Pohl, Leona Bauer, Ioanna Mantouvalou, Ulla Simon, Claudia Fleck

**Affiliations:** 1 Technische Universität Berlin, Faculty III Process Sciences, Institute of Materials Science and Technology, Fachgebiet Werkstofftechnik/Chair of Materials Science & Engineering, Berlin, Germany; 2 Technische Universität Berlin, Faculty III Process Sciences, Institute of Biotechnology, Chair of Applied and Molecular Microbiology, Berlin, Germany; 3 Helmholtz-Zentrum Berlin, Berlin, Germany; 4 Technische Universität Berlin, Faculty II Mathematics and Natural Sciences, BLiX, Institute for Optics and Atomic Physics, Analytical X-ray physics, Berlin, Germany; 5 Technische Universität Berlin, Faculty III Process Sciences, Institute of Materials Science and Technology, Chair of Advanced Ceramic Materials, Berlin, Germany; Leibniz-Institut fur Naturstoff-Forschung und Infektionsbiologie eV Hans-Knoll-Institut, GERMANY

## Abstract

Humanity is often fascinated by structures and materials developed by Nature. While structural materials such as wood have been widely studied, the structural and mechanical properties of fungi are still largely unknown. One of the structurally interesting fungi is the polypore *Fomes fomentarius*. The present study deals with the investigation of the light but robust fruiting body of *F*. *fomentarius*. The four segments of the fruiting body (crust, trama, hymenium, and mycelial core) were examined. The comprehensive analysis included structural, chemical, and mechanical characterization with particular attention to cell wall composition, such as chitin/chitosan and glucan content, degree of deacetylation, and distribution of trace elements. The hymenium exhibited the best mechanical properties even though having the highest porosity. Our results suggest that this outstanding strength is due to the high proportion of skeletal hyphae and the highest chitin/chitosan content in the cell wall, next to its honeycomb structure. In addition, an increased calcium content was found in the hymenium and crust, and the presence of calcium oxalate crystals was confirmed by SEM-EDX. Interestingly, layers with different densities as well as layers of varying calcium and potassium depletion were found in the crust. Our results show the importance of considering the different structural and compositional characteristics of the segments when developing fungal-inspired materials and products. Moreover, the porous yet robust structure of hymenium is a promising blueprint for the development of advanced smart materials.

## Introduction

A large number of fungi species populate the earth [1] and are found in a wide range of habitats [2]. Fungal species are classified into five divisions (so-called “taxonomic rank”), one of which are Basidiomycota. Within this species, white-rot fungi attack and degrade wood with an important environmental role in recycling dead trees. The white-rot fungus *Fomes fomentarius* (*F*. *fomentarius*) is a widely known species that lives preferably on birch and beech trees of the northern hemisphere. The fruiting bodies are hoof-shaped and of gray to brownish colour [3]. The most famous use of *F*. *fomentarius* as tinder required soaking in potassium nitrate [4,5] and has given it its colloquial name “tinder fungus”. In Romania and Hungary, it is still used for making textiles such as hats and vests [6]. It has also been used for various medical purposes such as antiseptic wound dressings [7]. Nowadays, *F*. *fomentarius* is one of few fungal species investigated to produce novel environmentally friendly materials for packaging, building, or insulation applications [8,9].

The fruiting body of *F*. *fomentarius* consists of several parts. Near the trunk is the mycelial core. Below the mycelial core is the hymenium. The hymenium is the spore-bearing part that faces the ground and has a honeycomb-like structure [10,11]. Both, the mycelial core and hymenium, are surrounded by the trama in the upward-facing area. The trama in turn is enclosed by a hard crust [12]. All of these segments are made of hyphae [13], filamentous fungal cells. Together, the filamentous hyphae form a network called mycelium. In general, fungi can consist of up to three different types of hyphae, starting with generative hyphae, which are present in all filamentous fungi. Depending on the species, generative hyphae may develop into skeletal hyphae and binding hyphae, in which case the system is termed trimitic, as for *F*. *fomentarius* [14]. While generative hyphae may be branched and contain septa that divide the filament into blocks, skeletal and binding hyphae have no septa and thick cell walls. Skeletal hyphae usually show a preferred orientation and binding hyphae are always branched [14]. In *F*. *fomentarius* generative hyphae are 2–4 μm in diameter, skeletal hyphae 3–8 μm, and binding hyphae 1.5–3.0 μm [15]. However, the distribution of the different hyphal types in the different segments of the fruiting body is not precisely known.

Like plant cells, fungal cells are surrounded by a cell wall. The fungal cell wall is a dynamically changing, porous composite consisting of chitin, glucans, and a variety of cell wall proteins [16]. Chitin is the structural backbone of the fungal cell and accounts for 1–15% of the cell wall biomass. It is composed of β-(1,4)-linked N-acetylglucosamine subunits and is therefore similar to cellulose, except for the side chain containing an acetyl group. The chitin homopolymer forms antiparallel β-sheets that are stabilized by intramolecular hydrogen bonds due to the acetyl group [17,18]. Natural chitin in fungal fruiting bodies is partially deacetylated, which means that different proportions of chitin and chitosan, that is chitin with a degree of deacetylation above 75%, are found [19].

The most abundant polymer in the fungal cell wall is glucan (30–80% of the cell wall mass [20,21]). β-1,3-glucan is covalently bound to chitin [22]. In addition, three strands of β-1,3-glucan are formed into a triple helix and held together by hydrogen bonds. In the division Basidiomycota, β-1,3-glucan and β-1,6-glucan together form an elastic and highly branched network [17,23,24].

In most cases, mycelial samples are studied with constant culture conditions, but different from the natural habitat [25–27]. This automatically leads to lower cell differentiation. Neither different hyphal types nor the different segments of a fruiting body are considered. For naturally grown specimens, authors usually do not distinguish between the different segments, e.g. hymenium and trama [28]. A recent study revealed significant differences between the microstructure, porosity, and the relative amounts of glucan and chitin of the crust, trama, and hymenium of *F*. *fomentarius* [11]. Unlike the trama and the hymenium, the crust consists of a dense network of hyphae and extracellular substances. Most striking is the occurrence of α-glucan exclusively in the crust. The authors propose an adapted model of the cell wall in which the crust is enriched in rigid lipids, proteins, and α-glucan compared to the hymenium and trama [11].

To date, most studies have examined the biology of fungi in terms of growth, spore shedding, metabolism, and the complex composition of the fungal cell wall [13,17,18,23,29–33], but only a few have addressed the mechanical properties. The mechanical properties were shown to depend on the hyphal systems [34]. For the hymenium of the trimitic system (three hyphal types) of *Ganoderma lingzhi*, an average compressive strength of 6.3 MPa was reported, which is 50 times higher than the value measured for the monomitic samples (one hyphal type). The (trimitic) hymenium of *F*. *fomentarius* showed values in a similar range for compressive loading parallel to the honeycomb structure of 4 to 6 MPa [10,11]. Much lower values were measured in the transverse direction (0.6 MPa) [10]. Delamination and buckling of the honeycomb structure were observed as predominant failure mechanisms [10,11]. The compressive strength of the trama is reported with 0.6 to 1.8 MPa [11]. Further, the chitin content has been shown to influence the mechanical strength. For instance, the tree bracket fungus *D*. *confragosa* has a much lower chitin content compared to common mushrooms, which drastically decreases its tensile strength [35].

In conclusion, little is known about the correlation between composition, structure, and mechanical properties of the segments of the fruiting bodies of *F*. *fomentarius*. We therefore performed a comprehensive study on the structural and chemical characteristics of the mycelial core, the trama, the hymenium, and the crust of the wood-degrading fungus *F*. *fomentarius*. By combining mechanical testing with high-resolution 2D and 3D imaging and several chemical analysis methods, we elucidate the influence of compositional and structural parameters on the mechanical properties. Our results contribute to the overall understanding of the hierarchical structure of white-rot fungi, such as *F*. *fomentarius*, and its influence on the macroscopic properties. Ultimately, this will help us to efficiently design future fungal-based materials.

## Results

### Macro- and microstructure

**Fig 1:**
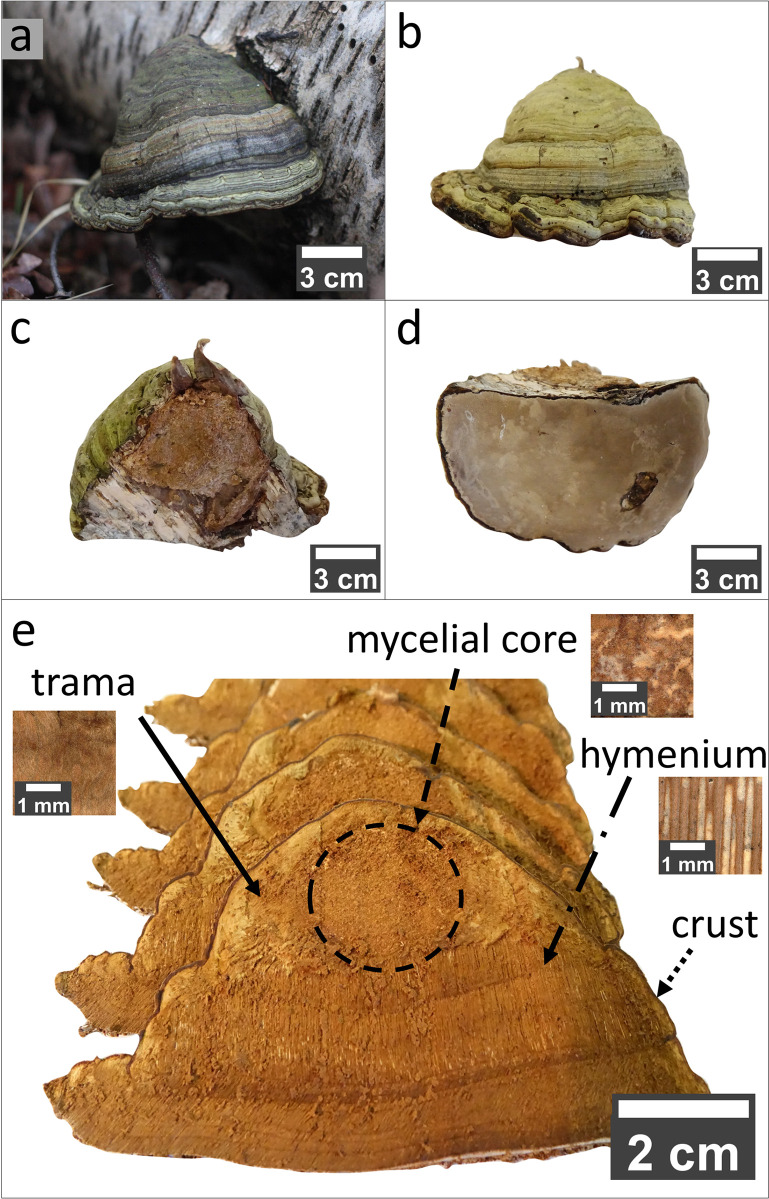
Macroscopic overview of the specimen of *F*. *fomentarius* under investigation. **a** Tinder fungus on a fallen birch with a typical hoof shape and grayish coloration. **b**, **c,** and **d** show different orientations of the specimen in **a**. While **b** shows the front side and **c** the back side connected to the tree, **d** shows the underside facing the ground in the natural environment. **e** shows slices of the specimen in **a**. The slices contain different parts of the fruiting body. The circle marks the mycelial core (dashed arrow). Around the mycelial core is the trama (full arrow). Below the trama and mycelial core are tubes in the mm range called hymenium (dash-dotted arrow). The entire fruiting body is surrounded by a rigid outer layer, the crust (dotted arrow).

An *F*. *fomentarius* fruiting body was collected from a fallen birch tree in the Grunewald forest, Berlin. The investigated specimen is shown in Fig 1A–1D. The four different segments, trama, hymenium, mycelial core, and crust can clearly be distinguished (Fig 1E).

**Fig 2:**
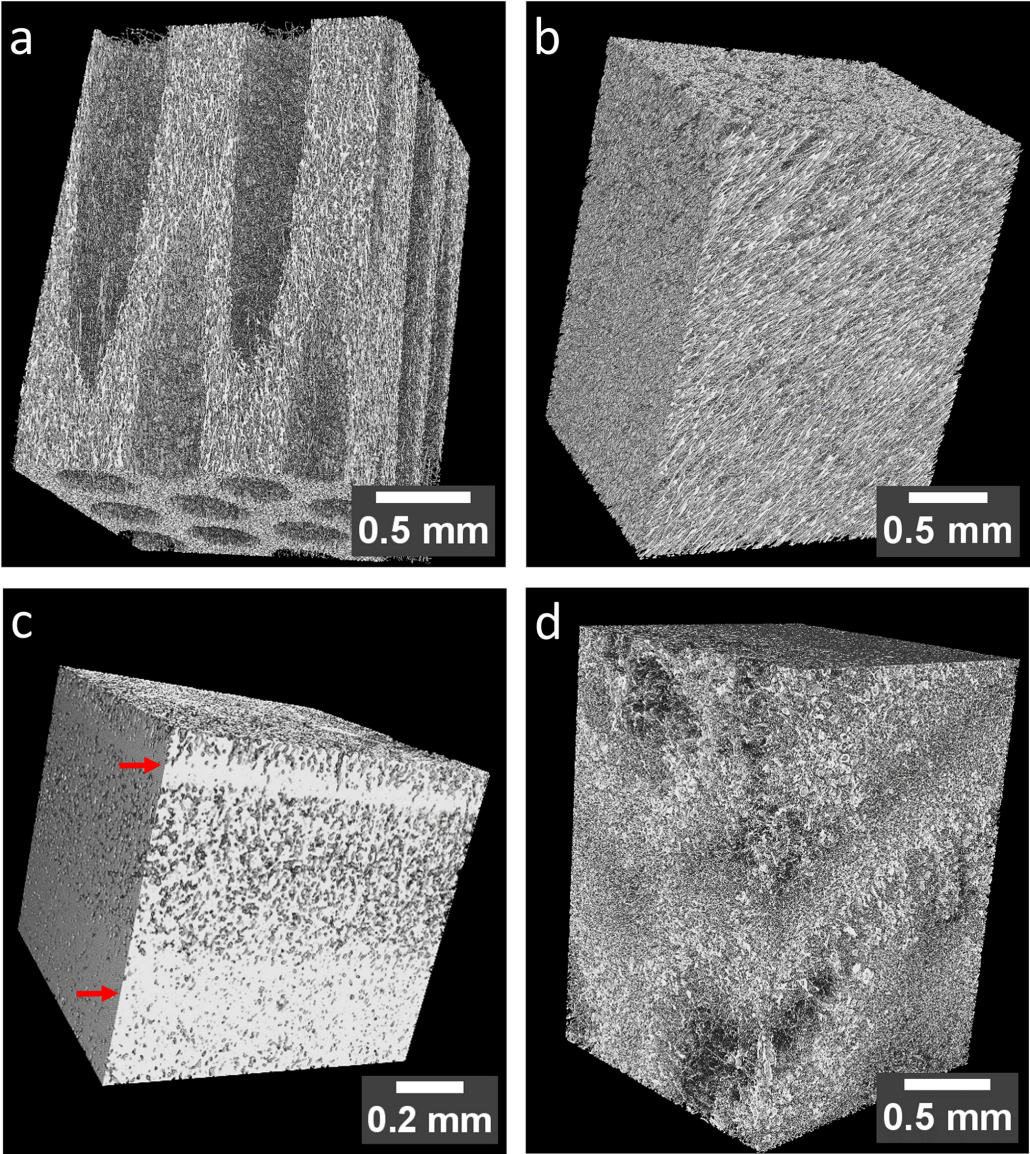
Microstructure by μCT of the four segments of *F*. *fomentarius*. **a** The volume reconstruction of μCT data shows the anisotropic structure of the hymenium. Elongated tubes traverse the mycelium. The holes are arranged in parallel, resulting in a transversely isotropic structure. At the micrometer scale, the structure consists of hyphae arranged predominantly parallel to the holes. Loose hyphae can be seen within the holes. **b** The volume reconstruction of the trama shows a uniform distribution of hyphae over the millimeter length scale. The hyphae are oriented parallel to each other. In **c** the crust shows fused, dense hyphae at the bottom, a less dense region in the middle, and a thin dense layer (arrows) before the outermost rough surface layer. **d** shows the mycelial core, a patchwork of regions of lower and higher density and different kinds of cells namely fungal but also wood cells.

**Fig 3:**
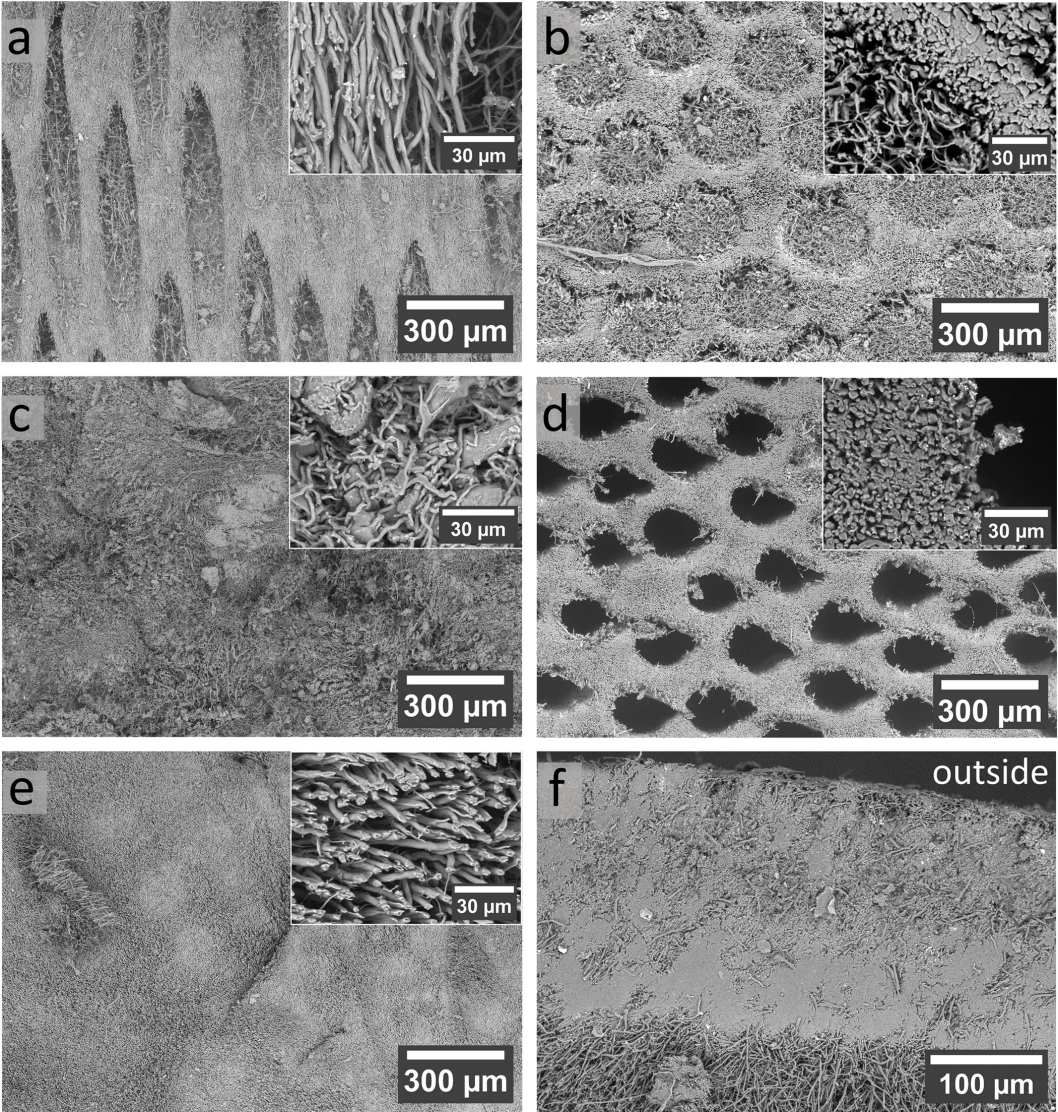
SEM images show the microstructure of different segments of *F*. *fomentarius*. **a**, **b,** and **d** show the hymenium in parallel and in transverse direction. The mycelium has a distinct transverse isotropic structure and forms elongated tubes. **b** and **c** show that the hymenium near the mycelial core has holes filled with generative hyphae, whereas the holes in new layers, near the bottom of the fruiting body, are empty. In **c**, dense and less dense areas are seen, with mainly generative hyphae and probably wood cells forming the mycelial core. In **e**, the trama is seen, which is composed largely of skeletal hyphae forming a uniform mass with some denser areas. In **f**, the thin dense part of the crust can be seen. The hyphae appear to be compressed and form a dense protective layer.

Figs 2 and 3 provide a comprehensive view of the segments of the fruiting body, presenting insights from 3D reconstructed tomography data and scanning electron microscopy (SEM). The hymenium is characterized by elongated, parallel tubes facing the ground with diameters around 200 μm, resulting in a transversely isotropic structure [10,11]. The tube walls consist of a network of hyphae, and to some extent, these hyphae align with the long axes of the tubes (Figs 2A and 3A). Within the tubes, a loose network of thin hyphae is found. Notably, Fig 3A and 3B offer additional insights into the hymenium, showing thick skeletal hyphae (⌀ 3–4 μm) forming the walls of these tubes. Within the tubes, thin hyphae (⌀ 0.6–0.8 μm), probably binding hyphae are found. The amount varies depending on the specific area within the hymenium (Fig 3B and 3D). The hymenium has a porosity of 76%. The trama appears homogeneous on the sub-millimeter scale, with hyphae exhibiting a clear preferred orientation, running parallel to each other at the micrometer scale (Fig 2B). The porosity in this segment is slightly lower at 70% compared to the hymenium. SEM images (Fig 3E) of the trama in the sub-millimeter range reveal denser areas with no particular arrangement. Section traces are evident, where hyphae appear torn off. At the microscale, mainly skeletal hyphae are visible, with some areas containing probably binding hyphae, adding complexity to the microstructure. The mycelial core appears the least organized within the fruiting body. It consists of a patchwork of regions with varying porosity (Fig 2D). The hyphae in this area are thinner than skeletal hyphae (⌀ 1–2 μm), curved, and lack a preferred orientation, which indicates generative hyphae. Additionally, larger cells resembling wood cells are observed (Fig 3C). The porosity is 61%. Figs 2C and 3F show the crust’s structure within the fruiting body of *F*. *fomentarius*. The upper side of the crust faces outward when observing the entire fruiting body, while the lower side is adjacent to the trama. The crust is constructed from layers of compressed hyphae. Near the trama, there is a dense layer of strongly compressed hyphae, followed by a less dense layer. Toward the outside, a second, thin, dense layer is superseded by the rough, outer surface layer. The porosity of the crust is 19%.

### Chemical composition

**Fig 4:**
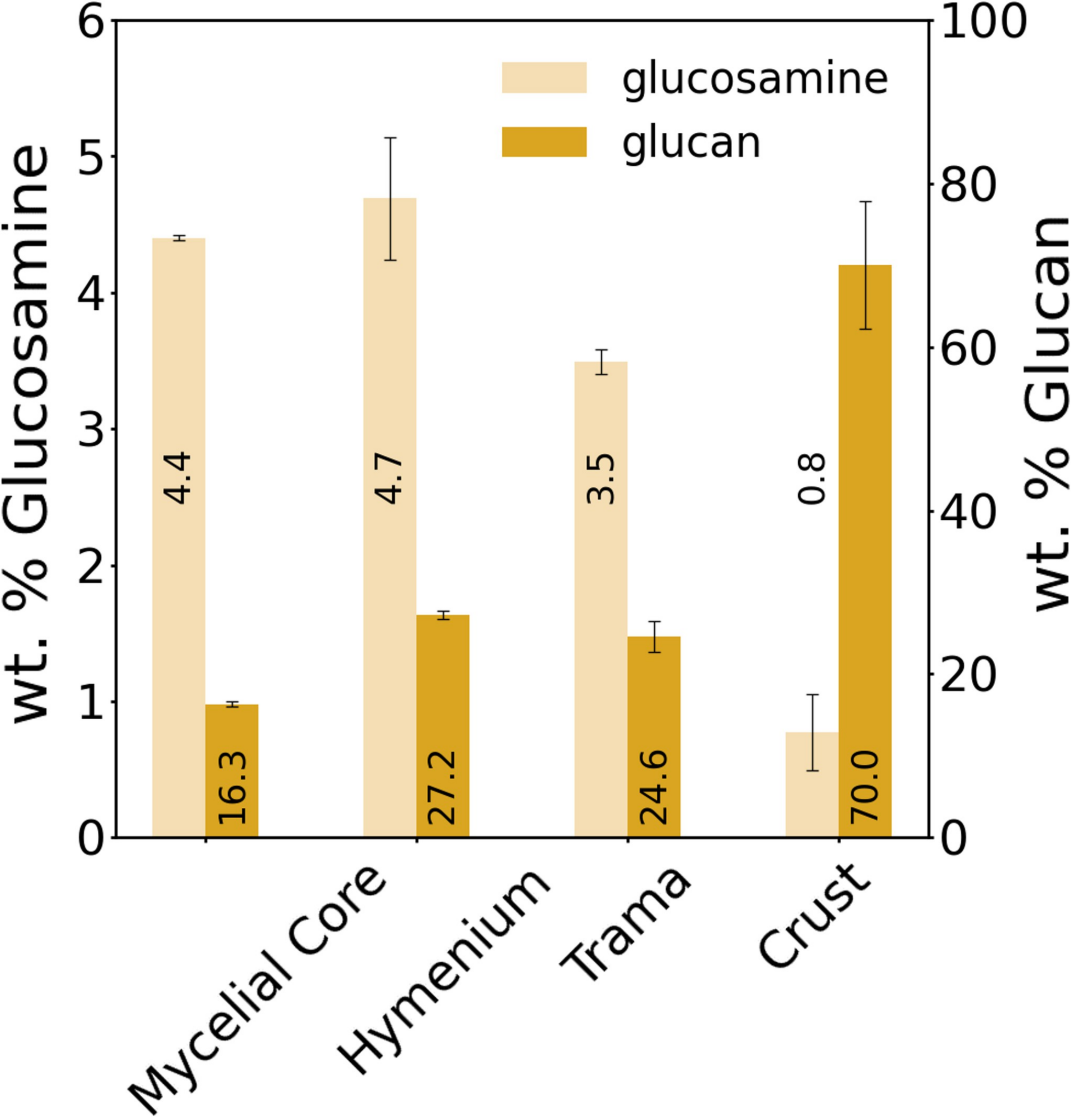
Chitin/chitosan and glucan content. The amount of glucosamine (wt.%) (the chitin/chitosan monomer) and glucan (wt.%) was measured by chitin and glucan assays, respectively. Mean values and standard deviations are given.

Chitin and glucan are the fundamental constituents of fungal cell walls. To quantify the two polysaccharides, we conducted chitin and glucan assays (Fig 4). Glucosamine is the monosaccharide constituting chitin and chitosan, so measuring its quantity is equivalent to assessing the chitin and chitosan content in fungal structures. Significant variations in chitin/chitosan and glucan content across the different segments have been observed, with higher chitin/chitosan levels (5 wt.%) in the hymenium and nearly negligible chitin/chitosan content in the crust (<1 wt.%). Regarding the glucan content, the crust exhibited the highest concentration (70 wt.%) and lowest in the trama (16 wt.%).

**Fig 5:**
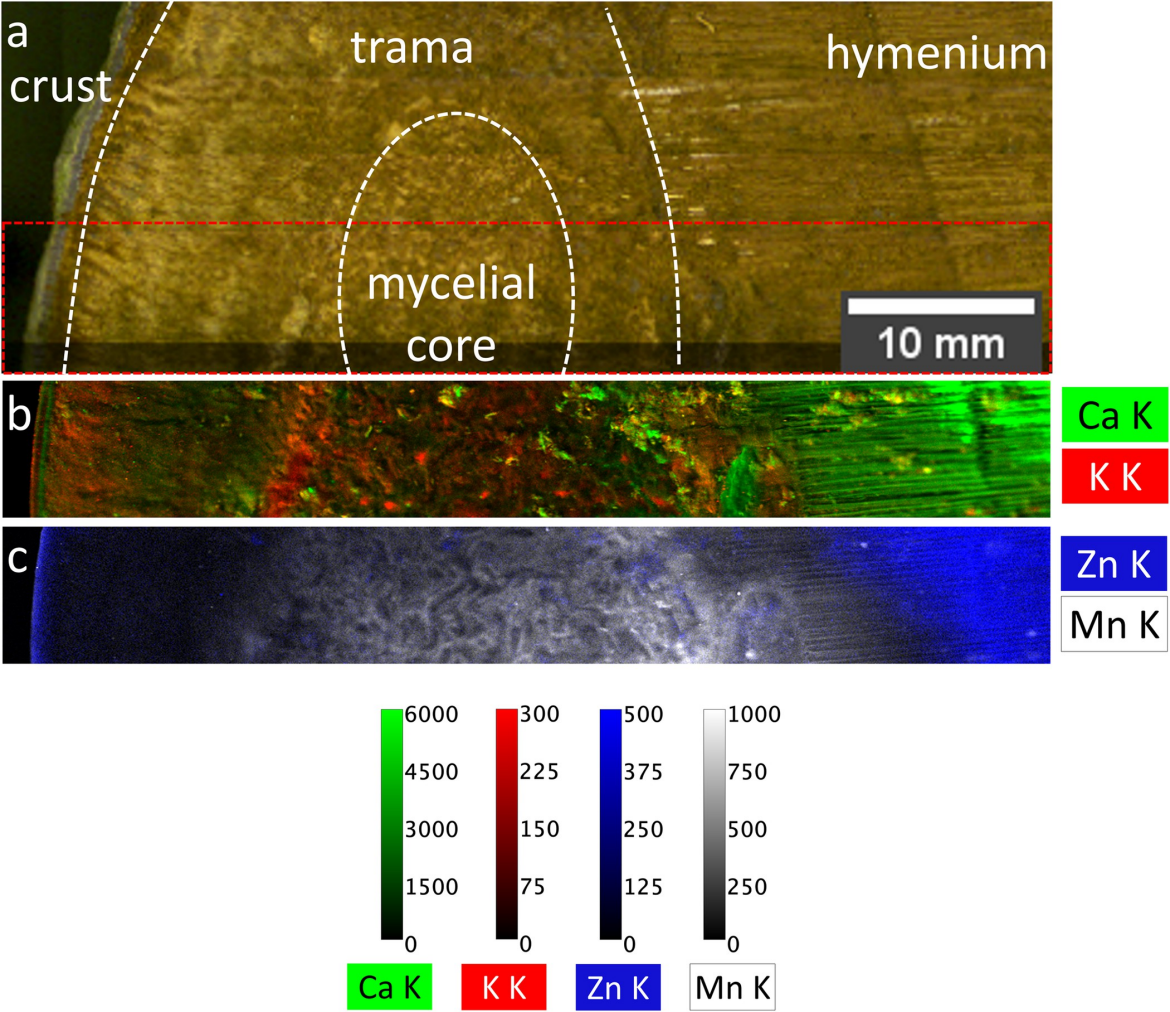
Micro X-ray fluorescence (μXRF) of a slice of *F*. *fomentarius*. All four segments are shown in a. On the left side, the crust joins the trama surrounding the mycelial core. On the right side, the hymenium follows. The area marked by the red square was examined by μXRF. b shows potassium (K) and calcium (Ca) distributions, and c shows zinc (Zn) and manganese (Mn) distributions. The color scales give the measured intensities in counts per second of the fluorescence signal.

**Fig 6:**
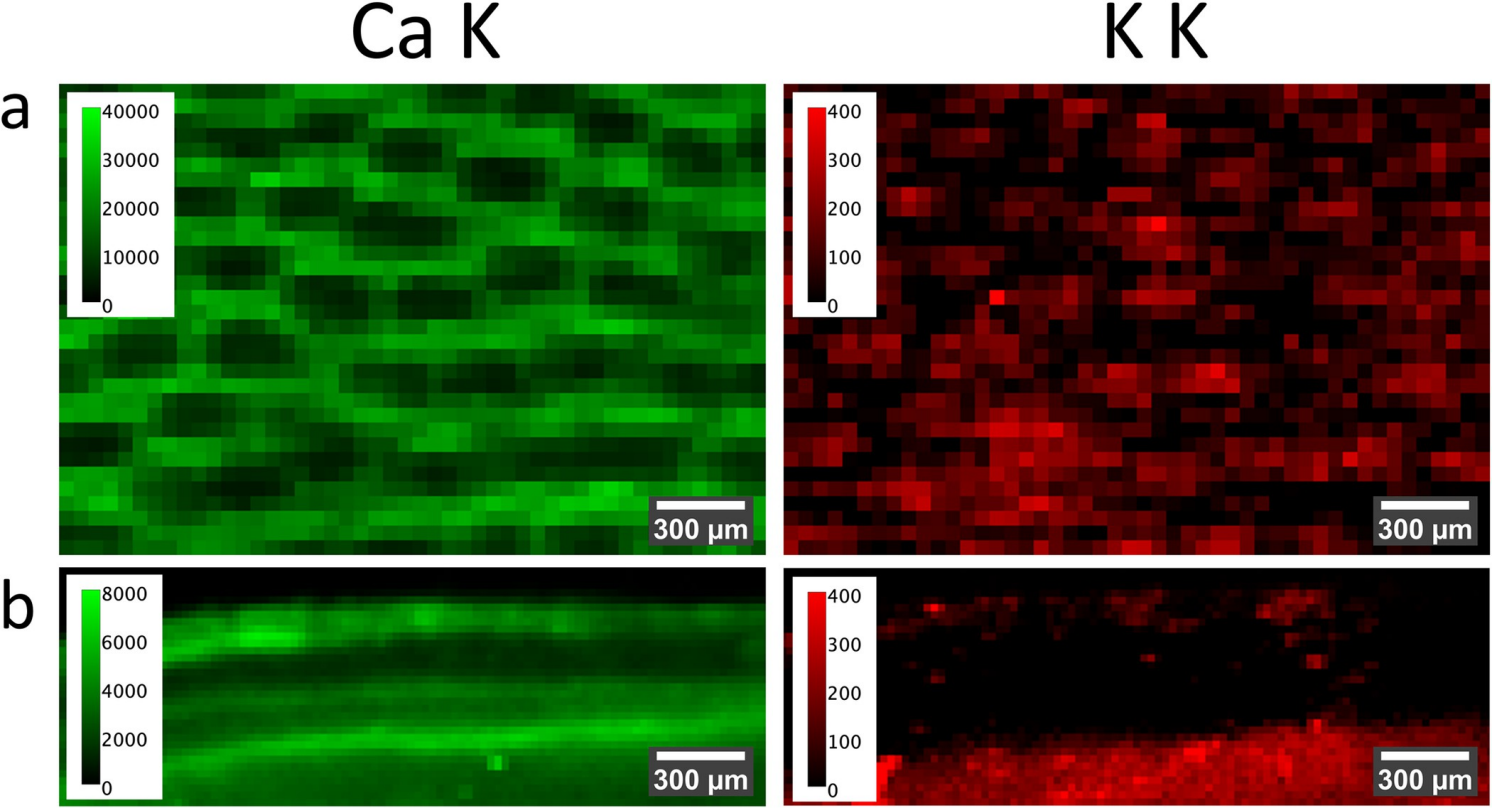
Magnification of μXRF of hymenium and crust. Zooming in on **a** the hymenium (tubes facing the observer) and **b** the crust (cross-section) for the Ca K and K K distributions. The color scales give the measured intensities in counts per second of the fluorescence signal.

Fig 5 shows composite images of the 2D distributions of the intensities of the K fluorescence lines of calcium (Ca) and potassium (K) (Figs 5B and 6), zinc (Zn), and manganese (Mn) (Fig 5C) derived from micro X-ray fluorescence (μXRF) maps across the segments of the fruiting body. The hymenium exhibits enhanced Ca (Fig 6A) and Zn signals, whereas the Mn K fluorescence intensity is predominantly enhanced in the mycelial core. Moreover, increased Ca K and K K intensities are detected within the mycelial core and the trama. A more detailed examination of the cross-section of the crust (Fig 6B) reveals that Ca is distributed across several layers within the crust. Conversely, K is nearly absent in most parts of the crust (Fig 6B), while the Zn signal is notably elevated (Fig 5C).

**Fig 7:**
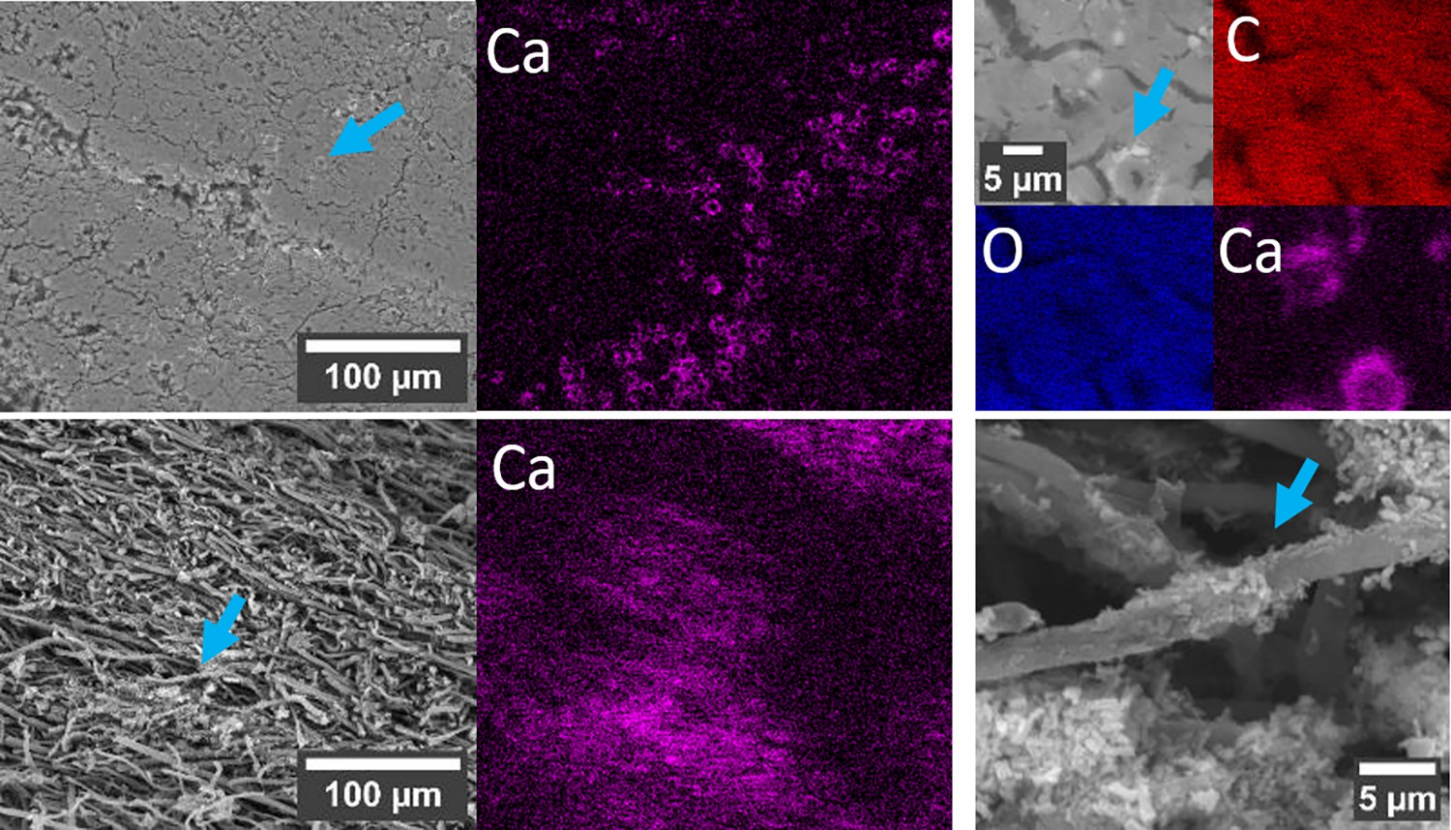
SEM images and corresponding SEM-EDX calcium maps of the crust and hymenium. **a** Inside of the crust, viewed parallel to its outer surface, revealing circular patches rich in calcium. **b** Closer view of the hypha indicated by the arrow in **a**, highlighting the brighter regions surrounding the hypha, where Ca concentrations are notably higher. **c** Hymenium (view parallel to the tubes) showing elevated Ca levels, mainly in **d** crystals situated between the hyphae.

The observation of elevated Ca K fluorescence intensities in the hymenium and the crust led us to a closer examination via SEM-EDX. Fig 7A and 7C show an SEM survey of the crust and the hymenium, respectively, and the corresponding Ca distributions. Higher magnifications (Fig 7B and 7D) reveal the presence of crystals surrounding the hyphae. EDX mapping in the region shown in Fig 7B reveals a slight depletion of C in the area where Ca is found, while O is distributed homogeneously. This higher Ca content hints to the presence of calcium oxalate crystals.

**Fig 8:**
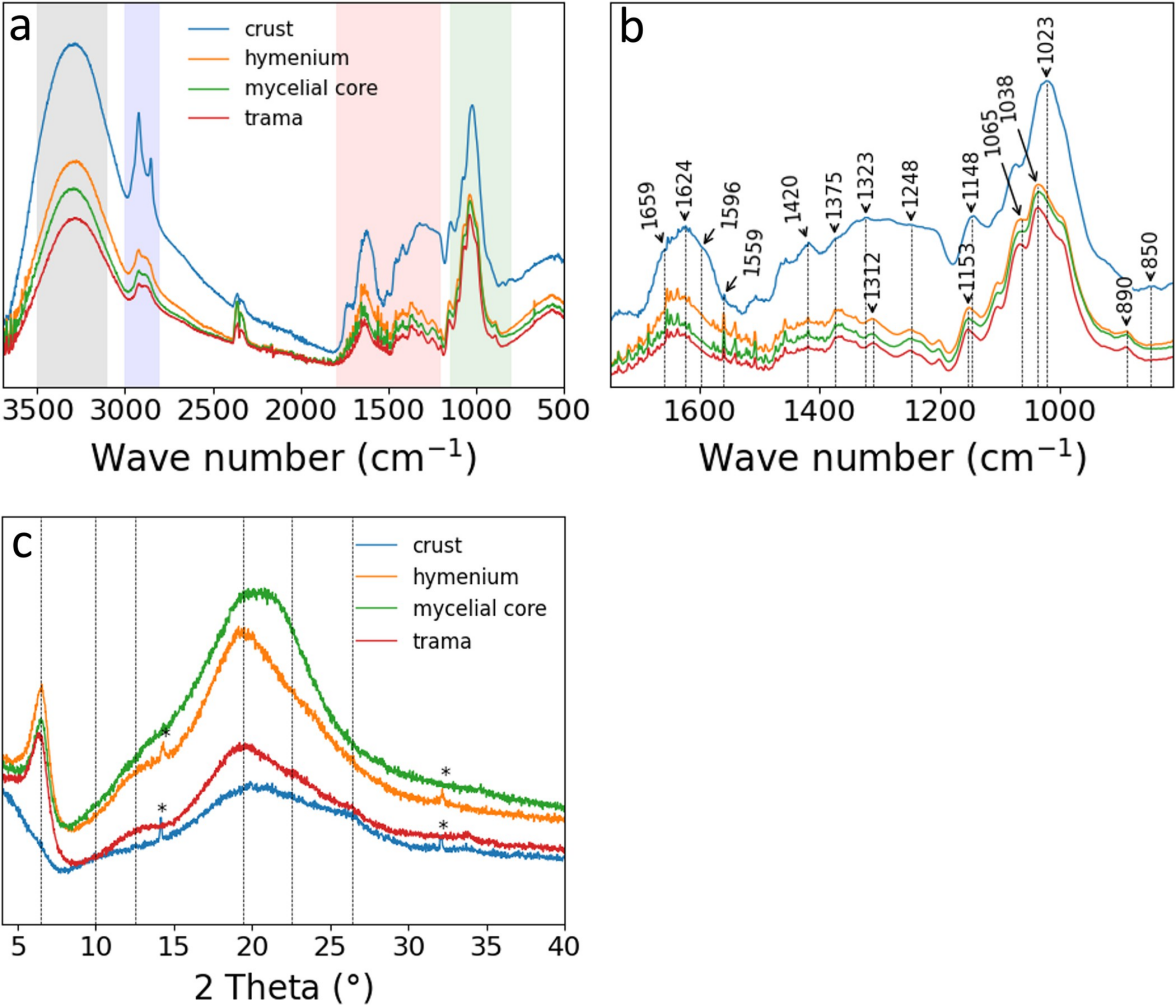
FTIR–ATR spectra X-ray diffraction patterns of the crust, hymenium, mycelial core, and trama. **a** Full FTIR spectrum; the colored regions show: -OH and -NH stretching bands (grey), lipid region (blue), amides and proteins (red), and polysaccharides like glucans (green); **b** fingerprint region. **c** XRD patterns of the four segments.

Fig 8A and 8B show absorption spectra obtained by ATR-FTIR. All spectra show a broad and strong absorption band between ~3600 cm^-1^ and ~3000 cm^-1^ (grey frame), characteristic of the -OH (common to polysaccharides) and -NH stretching bands. In addition, the stretching of -CH_2_ and–CH_3_ is pronounced at 2854 cm^-1^ and 2924 cm^-1^ (lipid region, blue frame), where the 2854 cm^-1^ peak is more pronounced for the crust spectrum. In the fingerprint region (red frame), the stretching vibration modes of amide I between 1660 cm^-1^ and 1620 cm^-1^ indicate α-chitin [36,37]. Additionally, amide II and amide III bands are characteristic of fungal material [27,38]. Here, the crust spectrum differs from the other spectra indicating a higher degree of deacetlyation of chitin [39–41]. The bands at lower wavenumbers (1200 cm^-1^–800 cm^-1^, green frame) can be attributed to polysaccharides, such as glucans, with a maximum at ~1023 cm^-1^ for the crust indicating α-glucan, and a maximum at ~1038 cm^-1^ for the hymenium, trama, and mycelial core indicating the presence of β-glucan [27,40,42,43].

The XRD patterns of the four segments are shown in Fig 8C. In general, the amount of crystallization of the polysaccharides is comparatively low, resulting in diffraction curves with broad reflections. The X-ray diffraction patterns of hymenium, mycelial core, and trama show a well-resolved reflection at 2θ ~ 6.5° indicative for β-glucan [37], followed by a broad and large reflection with overlapping peaks from 2θ ~ 10° - 35° (2θ ~ 12.5°, 2θ ~ 19.3°, 2θ ~ 22.5°, 2θ ~ 26.4°), resembling chitin or the glucan-chitin complex [19,36,44–48]. The diffraction pattern of the crust differs, however. The reflection at 2θ ~ 6.5 is not pronounced, and a reflection around 2θ ~ 10° is visible, which might indicate α-glucan but the reflection might also be shifted due to deacetylation of chitin or incorporated water molecules [41,49–52]. Further intensities and positions of the overlapping reflections are similar to the patterns of the other three segments (2θ ~ 19.3°, 2θ ~ 22.5, and 2θ ~ 26.4°), resembling chitin or the glucan-chitin complex [19,36,44–48]. The crust and the hymenium show sharp but small reflection at 2θ ~ 14° and 2θ ~ 32° most probably calcium oxalate hydrate [53–55].

### Mechanical properties

**Fig 9:**
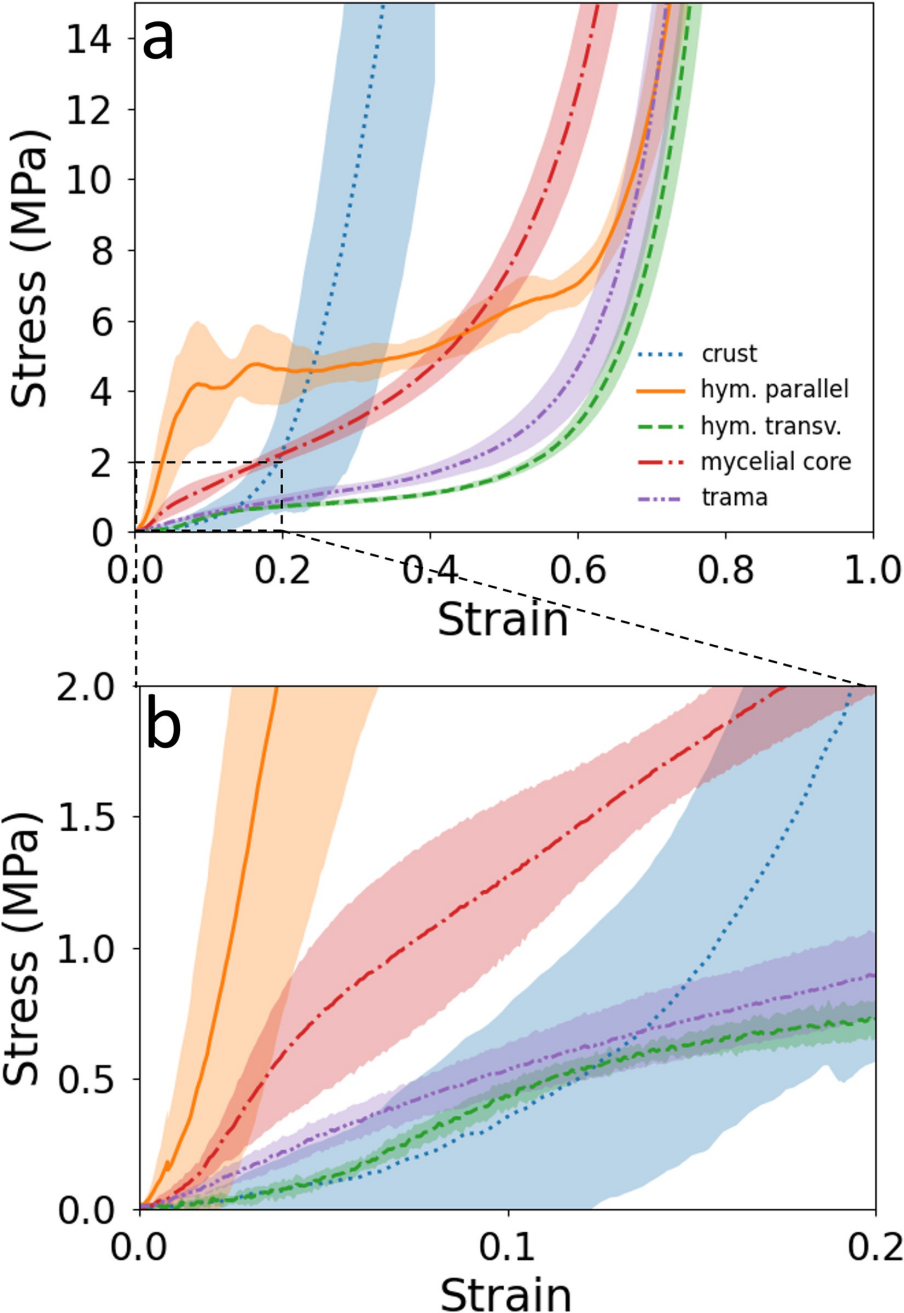
Stress-strain curves from compression tests on cube-shaped specimens from the four segments of *F*. *fomentarius*. **a** complete curves up to densification; **b** magnified view of the initial part.

Fig 9 shows the results of compression tests up to the densification range of cubes extracted from the four segments of the *F*. *fomentarius* specimen. Compression tests of the hymenium parallel (Fig 11B, orange arrow) to the elongated tubes show the typical deformation behaviour of an elastomeric honeycomb [10,56]. After a quasi-linear increase in stress, a stress plateau is observed. At a strain of 0.6, the stress increases as densification sets in. The plateau stress of 4.77 MPa (see Table 1) is 1.5 times the value of the mycelial core. In contrast to the hymenium in parallel compression, the stress in the mycelial core increases slowly at first (Fig 9B). However, after only a small amount of strain, the stress increases. In contrast to the hymenium, the structure of the mycelial core leads to a steady increase in stress and an earlier onset of the densification range at a strain of about 0.45. Compared to the mycelial core and hymenium in parallel compression, the trama shows a much lower stress response at the beginning and exhibits a plateau followed by a densification region typical for foams. In addition, the plateau stress is significantly lower at 1.22 MPa. However, the material is compressed for a long time without really being damaged. The hymenium compressed transversely to the elongated holes shows the lowest stress response in the beginning (Fig 9B) which is due to the compression of air in the transversely oriented holes. Accordingly, this leads to a plateau stress of 0.87 MPa. Thereafter, the material behaves similarly to the trama, with a plateau and densification setting in at a strain of 0.6. According to Fig 9A, the crust behaves differently compared to the other segments. After an inlet area, the curve rises steeply, suggesting that the crust is less accurately described by a foam.

**Table 1:** Results of the compression tests. The plateau stress *σ*_*pl*_ is the average stress between 0.2 and 0.4 strain. Accordingly, the confidential interval (CI) at 95% is given.

	hymenium parallel	mycelial core	trama	hymenium transverse
*σ*_*pl*_ [MPa]	4.77	3.20	1.22	0.87
95% CI	0.53	0.36	0.18	0.07

**Fig 10:**
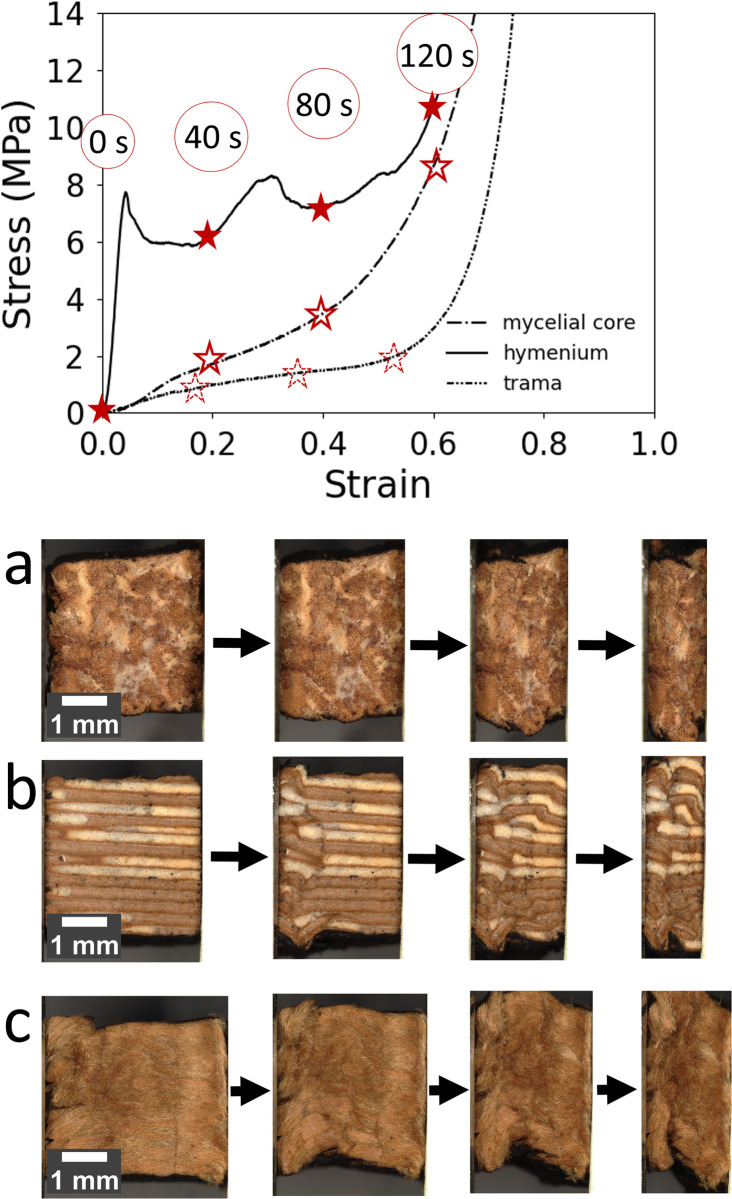
Representative stress-strain curves and optical micrographs. Cubes derived from the mycelial core **a**, hymenium **b**, and trama **c** at various time intervals during the experiment (marked by asterisks on the stress-strain curves). All specimens were loaded until densification.

To analyze the failure mechanisms, Fig 10 shows optical micrographs of the mycelial core, hymenium, and trama at different stages during the compression tests. The mycelial core and the trama show typical foam-like behavior, with buckling to the sides during the plateau region and before densification. The hymenium shows a different failure mechanism due to its honeycomb structure. The cell walls of the honeycomb are collapsing by buckling (40 s). The stress continues to rise until a second area collapses by buckling (80 s) and causes a drop in plateau stress. After complete collapse, the structure densifies (120 s).

## Discussion

Previous works on fungi focused on their biology in terms of growth, spore shedding, metabolism, and the complex composition of the fungal cell wall [13,17,23,29–32]. However, the mechanical properties have been mainly studied on fungal-based composites [8,57,58]. So far, only Müller *et al*., Porter *et al*., Wang *et al*., and recently Pylkkänen *et al*. report on mechanical tests on bulk fungal samples [10,11,34,59]. Here, we elucidate the differences in microstructure and chemical composition of the cell wall of all four segments of the fruiting body of *F*. *fomentarius*. to understand the differences in mechanical performance.

### Why is the hymenium so strong, and what is wrong with the trama?

The mechanical properties of the different segments of the fruiting body of *F*. *fomentarius* are diverse. The hymenium, mycelial core, and trama behave like different foams, but the crust compresses quickly and soon behaves like bulk material. The crust and hymenium, compressed parallel to the tubes, have the highest strength, the mycelial core is in the middle range, while the trama and transversely compressed hymenium have the lowest strength. This can be partially explained by porosity and microstructure. For instance, the porosity of the crust is by far the lowest, leading to higher strength and mechanical behavior that rather resembles that of a bulk material than a foam. In contrast, the hymenium has the highest porosity, and nevertheless a higher compressive strength than the mycelial core and trama. The honeycomb microstructure of the hymenium is a likely reason for the relatively high strength under parallel compression. This is supported by the fact that loading of the hymenium transversely to the tubes results in similarly low compressive strength as for the trama which lacks the honeycomb structural arrangement. Both the hymenium and the trama have a large number of skeletal hyphae with thick cell walls, but in the hymenium these are oriented along the honeycomb tubes and thus parallel to the direction of the compressive force. Others have partially attributed the higher strength of the hymenium to the presence of extracellular matrix [11], which we did not observe in our specimen. Further differences are the higher porosity and higher chitin/chitosan and glucan amounts that we find for the hymenium compared to the trama. In contrast, Pylkkänen *et al*. [11] showed larger glucan contents values and lower porosity for the trama (see Table 2). The presence of the chitin-glucan complex in hymenium and trama is also confirmed by XRD and FTIR measurements in this study. Therefore we attribute the better mechanical performance of the hymenium not only to its tubular honeycomb structure but also to its higher chitin/chitosan content. The lower chitin/chitosan content of the trama probably explains the lower strength but higher elasticity. This finding corroborates the results by Nawawi *et al*. who reported a decrease in tensile strength with decreasing amounts of chitin/chitosan [35]. We conclude that the trama acts as an elastic and fluffy, and thus probably protective sheath for the mycelial core.

**Table 2:** Influence of the geographical origin on the porosity, glucan, and chitin/chitosan contents of *F*. *fomentarius*: Comparison of our measurements with the results reported by Pylkkänen *et al*. [11].

	[11]			this work		
	hymemium	trama	crust	hymenium	trama	crust
**Porosity [%]**	70.9	47.7	11.5	75.5	70.1	19.1
**Glucan [%]**	38	69	55	27	25	70
**Chitin/chitosan [%]**	5	2	1	4.7	3.5	0.8

As compared to other studies, we measured similar compressive strength values of the hymenium (~5 MPa in this work as compared to 2–6 MPa [10,11,34]). The large range of the values may be due to different conditions of the natural habitat such as the availability of nutrients, natural fluctuations of biological samples from different individuals, and differences in sample preparation.

Of particular interest is the failure mechanism of the hymenium (Fig 10B), whose hollow tubes making up the honeycomb fail by regional plastic buckling, as also described by Müller *et al*. and later by Pylkkänen *et al*. [10,11]. As a result, local deformation bands are formed. In these, buckling leads to mutual support and densification. Consequently, stresses are transferred to the neighboring areas, and higher stresses are needed to lead to the collapse of the next area. Meanwhile, integrity is maintained in many other areas. With ongoing loading, increasingly more areas fail by buckling, leaving other areas intact. Catastrophic failure finally goes along with complete densification. This behavior likely allows for the task this segment has to perform: maintaining reproductive activities such as spore shedding.

### Strange mycelial core

In all hierarchical levels, the visual appearance of the mycelial core differs from both the hymenium and the trama. The strength of the tissue of the mycelial core is between the values exhibited by the other two segments. The fungal cells differ in thickness and curvature from the skeletal hyphae in the hymenium, and they resemble generative hyphae. Larger, roundish cells are also seen, which may be wood cells. This combination appears to result in a lower porosity compared to the hymenium and trama, and thus higher strength compared to the trama. In addition, the cells in the mycelial core have larger amounts of chitin/chitosan, comparable to the amount of chitin/chitosan in the hymenium, which likely is another factor contributing to the higher strength compared to the trama. Nevertheless the structure is weaker compared to the hymenium which could be attributed to the lack of the honeycomb structure and skeletal hyphae in the mycelial core.

### Revisiting the crust

As mentioned before the crust depicts by far the lowest porosity, which probably explains its mechanical response to compression, with a flat course in the beginning of loading and a steep rise in the further course of the experiment. Nevertheless, the crust does depict the lowest chitin/chitosan content and a high amount of glucan, which might indicate enhanced fracture toughness [35]. The SEM micrographs and EDX analyses reveal dense, seemingly compressed hyphae and calcium oxalate hydrate crystals. Additionally, in the 3D μCT images, a layered structure of two denser (arrows) and two less dense regions is seen, due to more and less compressed hyphae (Figs 2C and 3F). Curiously the biopolymer composition of the crust differs from other segments in the fungus, besides showing higher glucan contents, FTIR and XRD results suggest α-glucan in the crust. α- and β-glucans have also been observed in the crust by solid-state NMR, yielding a total glucan content of 55% of the total mass [11]. This value is lower than the 70 wt.% that we determined using the glucan assay. The different structure of α- and β-glucan has been suggested to influence the mechanical resistance of fungal cells [24,60,61]. While α-glucan occurs mainly in linear chains, β-glucan exhibits branched and unbranched molecular structures. So far, the role of α-glucan compared to β-glucan has attracted little research attention [24]. While many studies show the important role of β-glucan in the architecture and resistance of the fungal cell wall [24], only few studies report on the crucial role that α-glucan seems to play in maintaining cell shape and integrity or for drug sensitivity and conidia adhesion [60,61]. We may therefore conclude that the presumably higher content of α-glucan leads to better adhesion between the compressed cells in the crust, explaining the high strength. Additionally, α-glucan may therefore contribute to better resistance to environmental factors, such as wetness, UV radiation, or attack of invaders, which is an especially important quality of the outer crust. However, the study situation is not yet satisfactory in this respect.

Since the crust is a barrier of the fruiting body in contact with surrounding elements, such as water, the increased fracture toughness in the wet state could be useful. Nevertheless, the protective crust together with the padding abilities of the trama successfully shield the mycelial core and hymenium from the environment.

### What has that to do with the hierarchical structure?

The wood-degrading fungus *F*. *fomentarius* is an excellent illustration of the importance of hierarchical structuring in nature. In nature, one structure often has to serve several functions but is still composed of only a few constituents [62]. The fungal main constituent are hyphae. In the case of *F*. *fomentarius* we see three different types of hyphae, which build the macroscopic segments in the fruiting body differing optically, mechanically and chemically. The different arrangement of the hyphae result in honeycomb structures (hymenium) but also more foam-like (mycelial core, trama) or even solid (crust) structures at the sub-millimeter level decisively shaping the mechanical properties. In the μm-range, we see the different expressions of the hyphae types, which result in straight or curved, branched or unbranched, or even compressed cells. This level also contributes to the mechanical performance, as does the molecular level, which shows clear differences in cation distribution and biomolecule ratios. Here, the chitin content, the degree of deacetylation, and the content of α- and β-glucans could be decisive. Overall, the chemical composition shows the biggest differences in glucan and chitin/chitosan content and conformation compared to the hymenium, trama and crust. These diverse set of structures and thus properties ensures the survival of the fruiting body over several years.

### Understanding the metal ion distribution

2D elemental distributions of Zn and Ca show enhanced intensities in the hymenium. Calcium is an important trace element in eukaryotic cells and may be important for signaling in tip growth processes and the regulation of the hyphal orientation [63–66]. In detail, steep calcium ion gradients towards the growing tip are reported, with maximum levels at the end of the tip [67,68]. Zn is fundamental for cellular growth [65,69–71] and serves a catalytic function in fungi [65,72]. Ca and Zn ions not only play an important role in cell growth. In other organisms, they have been shown to contribute to self-healing mechanisms. For instance, Zn^2+^ in metalloprotein bonds allows byssal threads to stretch beyond failure and then regenerate [73], and Ca^2+^-stabilized networks contribute to viscoelastic recovery following repeated loading of caddisfly silk, protecting adhesive bonds from non-recoverable failure [74]. Comparable nanoscale mechanisms could come into play in combination with macroscopic failure mechanisms to protect the hymenium from instant catastrophic failure. Through μXRF, it becomes evident that the calcium content in the hymenium exceeds the Ca levels of the other segments. A more detailed examination using SEM-EDX and XRD confirmed the presence of crystals in the hymenium and in the crust, probably calcium oxalate hydrate [75], surrounding the hyphae and in the interstitial spaces (Fig 8C and 8D). It is worth noting that similar crystals were found in other wood-decaying fungal species [76] and many plants [77]. In plants it was shown that calcium oxalate hydrate crystals function in Ca regulation and protection against herbivory while also eventually enhancing mechanical stability [77–79].

The inhomogeneity of the mycelial core is reflected in the inhomogeneous distribution of Ca, Zn, and K. Interestingly, Mn K intensity is enhanced homogeneously, all over the mycelial core and in the area surrounding it. As Mn-dependent peroxidases play a role in degrading wood [65,80,81], Mn accumulation has also previously been found in the extracellular layer as well as in the degraded wood cell wall [80].

The crust has a layered structure, which is even more pronounced in the μXRF results (Fig 6B). The area facing the inside of the fungus exhibits several layers of higher Ca K intensity while it is depleted in K. We hypothesize that the higher Ca and lower K levels are related to the compression process during the formation of the crust. Ca ions indicate enhanced tip growth [63–66]. While K is also believed to play a role in hyphal growth [70], but K leakage has also been associated with the early stages of cell apoptosis [82–86]. Thus, processes of extended growth–indicated by high Ca levels–and cell death–indicated by K depletion, and visibly compressed cells, might run consecutively in time, which results in crust formation.

### How does all this help in fungal biomaterials research?

Various fungal species are used in fungal composites research. Besides the trimitic *Ganoderma lucidum* and the monomitic *Pleurotis ostreatus* systems [87–89], the trimitic *F*. *fomentarius* is another species of choice [8,38,90,91]. Differentiating the contributions of the various structures to mechanical strength, their structure-function relationships on the different length scales, and the interactions across length scales helps improving the engineering pathway to fungal composites with high strength, damage resistance, and durability. Currently, fungal biomaterials like mycelium composites show a relatively low compressive strength [8,56]. These materials mostly contain only one type of hyphae [8,58], and the hyphae are generally thin and form an anisotropic network. Firstly, the type of hyphal system has been shown to contribute to the mechanical strength in the range of a few MPa [34]. Similarly, our own results suggest that the natural fruiting body mainly consists of (stronger) skeletal hyphae, and, indeed, the strength of its segments is higher than the values measured for fungal composites. These findings therefore suggest a pathway of possible improvement in fungal composite mechanical properties by tweaking the hyphal type. Secondly, specifically in the hymenium, we observe a honeycomb structure with a preferred alignment of the hyphae in the tube walls. We hypothesize that this structural arrangement also contributes to the relatively high strength, yielding another possible line of optimization of fungal composite performance. A study from 2019 showed that the medium fungal mycelium is grown on indeed has an impact on e.g. hyphal density and hyphal diameter metrics [92]. This highlights that these properties, and thus the mechanical performance are tunable under laboratory conditions. Finally, the chitin/chitosan and the glucan contents strongly depend on the species as well as on the culturing conditions. They influence the mechanical properties even to a greater extent (in the range of tens/hundreds of MPa) as compared to hyphal type [35,92].

Furthermore, the architecture of natural fungi itself can serve as inspiration for the design of novel smart materials. The ability of the natural fungus to increase the compressive strength of some segments by a factor of 6 relative to others by applying a honeycomb structure to a fibrous material, and by additionally aligning these fibers in the direction of the honeycomb tubes demonstrates the inspirational potential of natural fungi.

## Conclusion

We employed 2D and 3D imaging, mechanical testing, and spectroscopic and chemical analysis to characterize not only the mechanical properties of different segments of the air-dried fruiting body of *F*. *fomentarius* but also the contribution of compositional and structural differences and the different hierarchical levels to these properties. Summarizing, we may draw the following main conclusions:

- The hymenium shows an interesting combination of high porosity and high strength (4.77 MPa), resulting from a honeycomb structure on the mesoscale. The trimitic hyphal system with a relatively high content in skeletal hyphae, and an elevated chitin/chitosan content in the hyphal cell wall contribute to strength on the microscale. A failure mechanism comprising plastic buckling and shear is exclusively found in the hymenium. Additionally, the occurrence of calcium oxalate hydrate crystals might influence the mechanical properties.
- The trama shows equally high porosity but low strength (0.89 MPa). Disordered skeletal hyphae and lower chitin/chitosan content in the hyphal cell wall might be the main reason for the low mechanical resistance.
- The mycelial core shows intermediate strength values (2.21 MPa), and lower porosity compared to the trama. Additionally, the form and thickness of the dominating hyphal type and the presence of woody cells determine the mechanical performance of the mycelial core.
- The crust shows the biggest deviations in structure and chemical composition compared to all other segments. The fungal cells seem compressed to a thick, dense layer with low porosity. Calcium accumulation and potassium depletion suggest a cycle of cell growth followed by cell apoptosis to build this rigid outer layer. Contrary to all other segments, α-glucan has been observed. As in the hymenium, calcium oxalate hydrate crystals are present.

The study of the hierarchical structure of *F*. *fomentarius* reveals large differences in the four macroscopically distinguishable segments. These differences should be kept in mind when designing novel fungal or fungal-inspired products. Furthermore, especially the porous but strong hierarchical structure of the hymenium is a promising blueprint for modern, smart, strong, yet light, and degradable materials.

## Methods

### Material preparation

**Fig 11:**
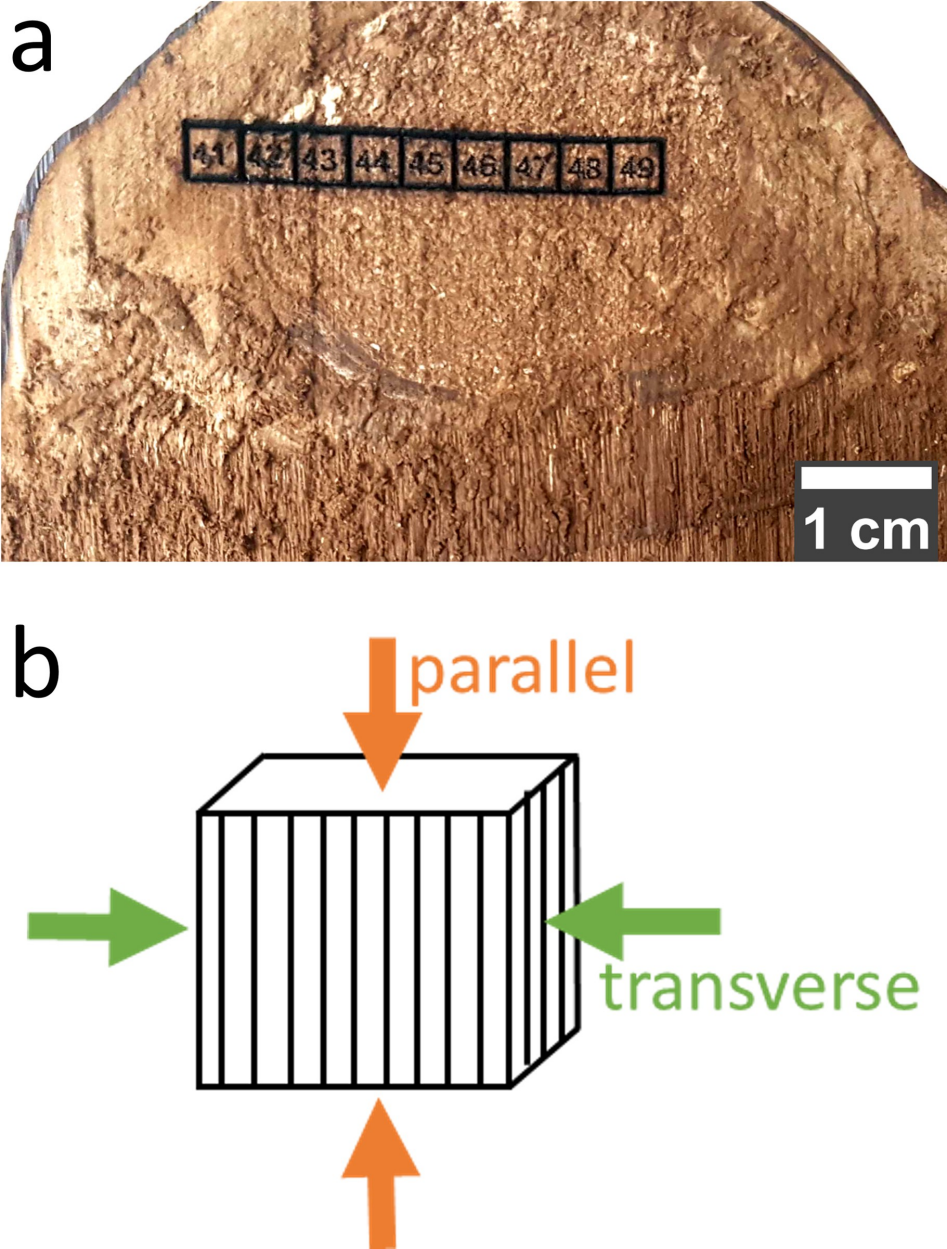
Laser-cut specimen and schematic of hymenium with compression directions. In **a**, a section with laser-cut square specimens is shown. The squares are numbered by carving with the laser, e.g. here in the segment of the mycelial core. **b** is a schematic representation of a cube specimen showing the possible compression directions (parallel and transverse) of the hymenium.

A tinder fungus (*F*. *fomentarius*) was collected from a fallen birch tree in Grunewald (Berlin, Germany). Note that the collection of fungi is legally permitted in Germany outside of nature conservation areas. The growth orientation of the fruiting body relative to the fallen tree (see Fig 1A) reveals that it developed after the tree fell. The specimen was ~13 cm by ~8 cm wide and ~12 cm high at its junction with the tree (Fig 1B–1D). After collection, the specimen was stored in a freezer at -18°C until preparation. The frozen fruiting body was cut into 3–4 mm thick slices using a band saw (Rekord, type SSF/420, Maschinenfabrik August Mössner KG, Germany) (Fig 1E) and air-dried for seven days. Compression cubes were cut with a laser cutter (Omtech, US) (Fig 11A) from three different segments, the mycelial core, the trama, and the hymenium (marked in Fig 1E). Further, pieces of the crust of approximately 2 x 2 x 0.5 mm^3^ in size were prepared with a scalpel.

To obtain powder for the elemental analyses, XRD and FTIR, several pieces of each of the four segments were ground with a ball mill (Retsch GmbH, Mixer Mill MM 400, Germany) for 2 minutes at 30 revolutions per second in a steel container with a steel ball.

### Microstructural characterization

Scanning electron microscopy (SEM; Phenom XL, Thermo Fischer Scientific, Netherlands) was performed to analyze the hyphal system of the different biological segments in *F*. *fomentarius*. SEM was used in the low vacuum, backscattered electron mode at a pressure of 60 Pa with an accelerating voltage of 10 kV. The outer faces of the laser-cut samples were taken off with a scalpel to eliminate the influence of the burned surface.

Furthermore, calcium distribution was analyzed using the low vacuum mode of the SEM (Quanta 400 FEG, FEI Company, United States) with an acceleration voltage of 20 kV energy-dispersive X-ray spectroscopy (EDX; EDAX Business Unit, AMETEK GmbH, Germany). The analysis was done on samples sputter coated with gold (30 mA, 2 x 15 s, Cressington 108auto, TESCAN GmbH, Germany) with the software EDAX Genesis (AMETEK GmbH, Germany).

### Microtomography

Micro-computed tomography (μCT) of laser-cut samples of the four different segments of *F*. *fomentarius* was performed at the microtomography setup of the ANATOMIX beamline at the synchrotron SOLEIL, Gif-sur-Yvette, France. A filtered white beam with a mean photon energy of about 36 keV was selected by implementing 20 μm-thin gold and 100 μm thin copper at an undulator gap of 5.5 mm. The detector was positioned 24 mm downstream of the specimen. An effective pixel size of 0.65 μm was selected. The detector consisted of a 20 μm-thin LuAG scintillator coupled via a 10× magnifying objective to a Hamamatsu Orca Flash 4.0 V2 scientific CMOS camera with 2048 × 2048 pixels each 6.5 μm wide [93]. Each tomography scan contained 2000 equidistant projection angles (0.09°) over a range of 180°, with an exposure time of 100 ms per projection radiograph. The scans were made on the fly, i.e., the sample kept rotating during image acquisition. Volumes were reconstructed using the open-source software PyHST2 (ESRF, Grenoble, France) [94]. Applying a Paganin filter [95] with a kernel length of 16.25 μm, the final resolution is estimated to be ~1.8 μm.

Data sets were binarised in Fiji/ImageJ (version 1.53s, May 2022) [96] and porosity was calculated using the Voxel Counter PlugIn. 3D images were created with the 3D Viewer PlugIn [97].

### Chitin & glucan assay

All reagents were purchased from Merck KGaA (Darmstadt, Germany) if not indicated otherwise. For chitin determination, ball-milled fungal samples were rinsed three times with 1 M NaCl and three times with water, following extraction of non-covalently linked cell wall components by heating for 5 min to 95°C in an extraction buffer (0.2% SDS, 5mM EDT, 100mM b-mercaptoethanol in MQ-H_2_O [98]). The washed fungal biomass was freeze-dried and 5 to 10 mg samples were subsequently hydrolyzed in 750 μl 6 M HCl at 100°C for 12 h. Samples were then neutralized by adding 750 μl 6 M NaOH. Subsequently, 40 μl of sample solution was mixed with 200 μl of chitin assay buffer (50 mM Na2SiO3, 600 mM Na2MoO4, 1.5 M CH3COOH, 30%(v/v) DMSO in MQ-H_2_O [99]) in a flat-bottom polystyrene 96-well MTP (Sarstedt AG, Germany) and incubated at 70°C for 30 min. The absorbance at 750 nm was read on an MTP spectrometer (GloMax, Promega GmbH, Germany). A standard curve from 0 to 2 mg/ml glucosamine (Sigma Aldrich/Merck, Germany) was used for calculating the glucosamine concentration in the fungal samples.

Glucan was determined by the method of Fortwendel *et al*. [100], using an aniline blue assay. Bead-milled fungal samples were washed with 0.1 M NaOH to remove soluble unbound glucan fractions and freeze-dried. 5 mg (dry weight) of each sample was resuspended in 250 μl of 1M NaOH, sonicated for 10 min in a water bath sonicator (VWR International GmbH, United States) and further incubated at 52°C for 60 min on a heated shaker (Eppendorf AG, Germany). 50 μl volumes of samples were transferred in triplicates to a 96-well microtiter plate (Sarstedt AG, Germany). A volume of 185 μl of aniline blue mix (0.067% aniline blue, 0.35N HCl, 0.98 M glycine-NaOH, pH 9.5) was added and the plate was incubated for 30 min at 52°C. After cooling to room temperature, fluorescence readings were acquired on a FLUOstar Omega microplate reader (BMG LABTECH, Germany) at 355 nm excitation and 520 nm emission. A standard curve was prepared from curdlan (Sigma Aldrich/Merck, Germany)

### Micro X-ray fluorescence analysis

Micro X-ray fluorescence (μXRF) maps were performed on an ~4 mm thick *F*. *fomentarius* slice (Fig 1e) using a modified Bruker Nano M4 Tornado equipped with a microfocus Rh X-ray tube [101]. The lateral resolution of the instrument amounts to about 30 μm for Fe Ka. Due to the energy-dependent attenuation of the exciting radiation and the detected fluorescence radiation, the information depth of the detected elements differs.

An overview map with a 50 μm step size and 500 ms measuring time, as well as detailed maps of the different areas with a 25 μm step size and 1 s measuring time at each measurement point, were performed. The overview map was measured under ambient conditions, the detailed maps were made using a 10 mbar vacuum environment. All measurements were performed with 50 kV acceleration voltage and 1 mA current. The collected full energy spectra at each measuring point of the maps are deconvolved using the M4 Tornado Software and the derived net peak intensities for several elements are shown as 2D distributions.

### X-ray diffraction

X-ray diffraction measurements were performed on a Bruker AXS D8 ADVANCE with a Bragg-Brentano geometry and a Lynx Eye 1D detector with CuKa1 radiation (0.154 nm) (Bruker, Germany) with a voltage of 40 kV and a current of 40 mA to study crystalline features on ball-milled samples.

### Fourier-transform infrared spectroscopy (FTIR)

FTIR spectroscopy in the ATR mode was carried out in a Vertex 70 (Bruker, Germany) over the range of 4000−400 cm^-1^ for the identification of distinct functional groups on ball-milled samples.

### Compression testing

For compression testing, cubic samples with an edge length of ~4 mm were cut as described in the material preparation section and loaded with a Kammrath & Weiss mechanical tester (Kammrath & Weiss GmbH, Germany). Force and displacement were measured with the inbuilt 500 N load cell with an accuracy of 0.5 N and the inbuilt displacement gauge with a range of +/- 6000 μm and an accuracy of 0.05 μm. The tests were performed in force control up to a maximum load of 490 N with a speed of 20 μm/s. The dimensions of each specimen were measured with calipers to calculate the stress *σ* and the strain *ε* from the recorded force-displacement curves. For all segments, specimens were tested parallel to the direction from the top to the bottom of the fungus. For hymenium, additional specimens were loaded orthogonal to this direction (Fig 11B). The mean curve of up to 8 experiments per segment and loading direction (for hymenium) and the confidence interval of 95% were calculated. The stress/strain curves were evaluated according to the German standard for compression testing of foams (DIN 50134). Accordingly, the plateau stress *σ*_*pl*_ was determined as the average stress between 20% and 40% strain. One specimen of each mycelial core, hymenium, and trama was compressed with the conditions above, and *in-situ* failure mechanisms were observed by using the record timer of a Keyence VHX-7000 (Keyence Deutschland GmbH, Germany) with a recording time of 20 s. Every 40 s are displayed.

## Supporting information

S1 Fig Calcium (Ca), potassium (K), zinc (Zn) and manganese (Mn) distributions in the four segments. **a** hymenium, **b** mycelial core, **c** trama, **d** crust, the scale on the microscopy image applies for the intensity maps, except for the crust where the scale at Mn K applies for the intensity maps.
(DOCX)
