# Supplementary material for: Hierarchical structure and chemical composition of complementary segments of the fruiting bodies of Fomes fomentarius fungi fine-tune the compressive properties
Source: PLoS One. 2024 Jun 13;19(6):e0304614. doi: 10.1371/journal.pone.0304614 (PMC11175439; doi:10.1371/journal.pone.0304614)
Supplement: S1 Fig — a hymenium, b mycelial core, c trama, d crust, the scale on the microscopy image applies for the intensity maps, except for the crust where the scale at Mn K applies for the intensity maps. (DOCX) [file pone.0304614.s001.docx]

**Supporting Information**

**Hierarchical structure and chemical composition of complementary segments of the fruiting bodies of *Fomes fomentarius* fungi fine-tune the compressive properties**

Sophie Klemm^1^, Carsten Freidank-Pohl^2^, Leona Bauer^3,4^, Ioanna Mantouvalou^3,4^, Ulla Simon^5^, Claudia Fleck^1*^

^1^Technische Universität Berlin, Faculty III Process Sciences, Institute of Materials Science and Technology, Fachgebiet Werkstofftechnik/Chair of Materials Science & Engineering, Str. des 17. Juni 135, 10623 Berlin, Germany

^2^Technische Universität Berlin, Faculty III Process Sciences, Institute of Biotechnology, Chair of Applied and Molecular Microbiology, Str. des 17. Juni 135, 10623 Berlin, Germany

^3^Helmholtz-Zentrum Berlin, Albert-Einstein-Str. 15, 12489 Berlin, Germany

^4^Technische Universität Berlin, Faculty II Mathematics and Natural Sciences, BLiX, Institute for Optics and Atomic Physics, Analytical X-ray physics, Str. des 17. Juni 135, 10623 Berlin, Germany

^5^Technische Universität Berlin, Faculty III Process Sciences, Institute of Materials Science and Technology, Chair of Advanced Ceramic Materials, Str. des 17. Juni 135, 10623 Berlin, Germany

*Corresponding author: claudia.fleck@tu-berlin.de


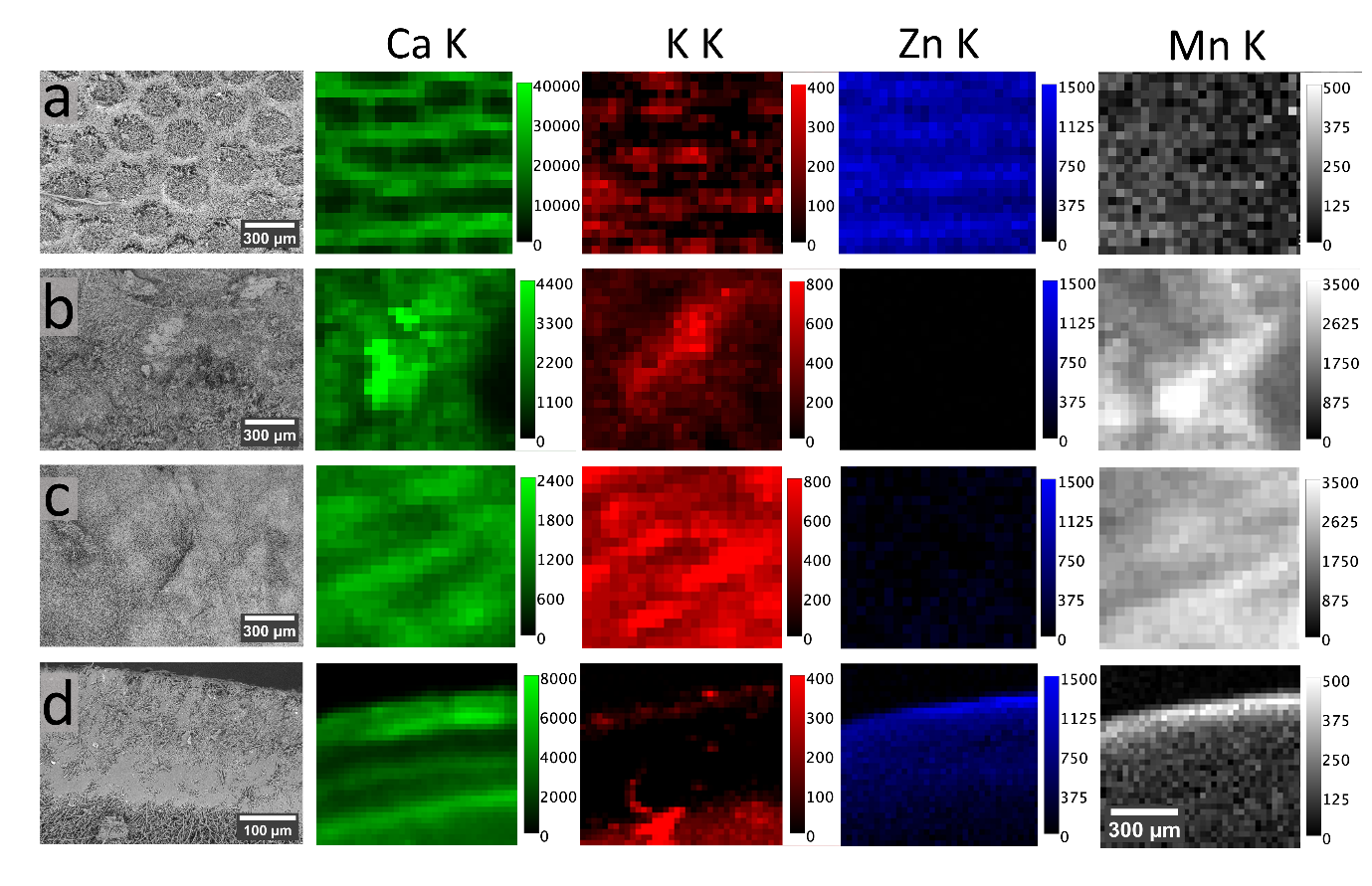


**S1 Fig. Calcium (Ca), potassium (K), zinc (Zn) and manganese (Mn) distributions in the four segments**

**a** hymenium, **b** mycelial core, **c** trama, **d** crust, the scale on the microscopy image applies for the intensity maps, except for the crust where the scale at Mn K applies for the intensity maps
